# The Geneva Convention

**Published:** 1899-10-28

**Authors:** 


### THE GENEVA CONVENTION.

In view of the intelligence that a considerable number of our wounded have been left in the hospital at Dundee, and of the anxiety which has been expressed in some quarters on the subject, we publish the exact wording of the Articles of the Convention which apply to the case in question:—

[Translation.]

Article I.

Ambulances and military hospitals shall be acknowledged to be neutral, and, as such, shall be protected and respected by belligerents so long as any sick or wounded may be therein.

Such neutrality shall cease if the ambulances or hospitals should be held by a military force.

Article II.

Persons employed in hospitals and ambulances, comprising the staff for superintendence, medical service, administration, transport of wounded, as well as chaplains, shall participate in the benefit of neutrality whilst so employed, and so long as there remain any wounded to bring in or to succour.

Article III.

The persons designated in the preceding Article may, even after occupation by the enemy, continue to fulfil their duties in the hospital or ambulance which they serve, or may withdraw in order to rejoin the corps to which they belong.

Under such circumstances, when those persons shall cease from their functions, they shall be delivered by the occupying army to the outposts of the enemy.

Article IV.

As the equipment of military hospitals remains subject to the laws of war, persons attached to such hospitals cannot, in withdrawing, carry away any articles but such as are their private property.

Under the same circumstances an ambulance shall, on the contrary, retain its equipment.

In Article VI. there is a provision that "those who are recognised, after their wounds are healed, as incapable of serving shall be sent back to their country." Other wounded, on the contrary, may be sent back on condition of not bearing arms again.

In regard to various questions which have been asked in Parliament, it is to be noted that it is definitely laid down in Article VI. that "wounded or sick soldiers shall be entertained and taken care of, to whatever nation they belong."

A very important question has been raised in regard to the status, under the Convention, of members of the purely combatant branches of the services when engaged in ambulance work. It has, for example, been stated that the Army Service Corps having become a combatant force, is now ineligible for work under the Red Cross, and an argument, founded on the same idea, was raised against the army medical officers being granted the purely military titles which they now enjoy. But reference to the wording of the Convention will show that any fear in regard to this matter is without foundation. As a fact no corps is ineligible to work under the Geneva Convention, provided its members shall have been authoritatively told off for the transport of or attendance on the sick and wounded in war, and that they wear the Red Cross badge. It is necessary, however, to bear in mind that, however fully the obligations of the Geneva Convention are recognised by the nations which have given in their adhesion to it, the provisions which it makes for the protection of the sick and wounded will always be very strictly limited to their intended objects. The following story was told some years ago by Professor Stevenson of Netley. After one of the fights about Sedan in 1870-71, the ambulances of a French division had fallen into the hands of the Germans, and Dr. Roth, of the German Army, was directed to ascertain if all those who had been taken prisoners were entitled to be considered neutral. Roth made no question as to the neutrality of the officer of the French "Intendance" (a combatant corps) who was in command of the ambulance transport, but he refused to consider as neutral that officer's soldier servant, whose only duty was to attend on his master, and not on the sick and wounded. The very essence of the Red Cross is that its use shall be restricted to those for whom it was intended by the Convention.

